# The Effect of Oral Nutritional Formula With Three Different Proteins on Type 2 Diabetes Mellitus *in vivo*

**DOI:** 10.3389/fnut.2021.680700

**Published:** 2021-09-21

**Authors:** Ye Jia, Yue Leng, Aliannys Lazára Puente Cruz, Chun Ling Bao, Bin Bao, Wenhui Wu, Peipei Wang, Ming Ma

**Affiliations:** ^1^Shanghai Ocean University, College of Food Science and Technology, Shanghai, China; ^2^Grupo Empresarial de la Industria Alimentaria, Havana, Cuba; ^3^East Hospital of Shanghai Sixth People's Hospital, Shanghai, China; ^4^Tarim University, Xinjiang Production and Construction Corps Key Laboratory of Protection and Utilization of Biological Resourses in Tarim Basin, XinJiang, China

**Keywords:** type 2 diabetes mellitus, oral nutritional formula, soybean protein, whey protein, silkworm pupa protein

## Abstract

Oral nutritional (ON) products are an effective way to treat patients with type 2 diabetes mellitus (T2DM) whose gastrointestinal functions are normal. The influence of ON formula prepared with three different proteins on T2DM was studied. The hyperglycaemic mouse model using a high-fat diet (HFD) combined with an intraperitoneal injection of streptozotocin (STZ) was used to simulate T2DM. The study was done for 15 weeks using seven groups of mice: control group (CG, normal mice, and normal food), non-treated group (BG, diabetic mice, and normal food), positive control group (PG, diabetic mice, and HFD), soybean protein group (SPG, diabetic mice, and HFD), silkworm pupa protein group (SPPG, diabetic mice, and HFD), whey protein group (LPG, diabetic mice, and HFD), and whey protein combined with silkworm pupa protein group (LCSSPG, diabetic mice, and HFD). The plasma levels of total cholesterol (TC), triglycerides (TG), low-density lipoprotein cholesterol (LDL-C), and high-density lipoprotein cholesterol (HDL-C) were analyzed on weeks 2, 10, 12, 14, and 15. The concentration of total protein (TP) and albumin (ALB) of the plasma was increased in SPG, SPPG, and PG comparing with BG (*p* < 0.05). The TC, TG, and LDL-C levels were decreased, and HDL-C level was increased in SPG, PG, SPPG, PG comparing with BG (*p* < 0.05). Blood glucose (BLG) levels were decreased 47, 34, 24, and 21% in SPG, LCSSPG, SPPG, and PG, respectively. While BLG was not significantly changed (*p* ≥ 0.05) in LG after 5 weeks of treatment. Overall, the data suggested that consumption of SP, SPP, LCSSPG Oral-formula may be beneficial for the treatment of T2DM.

## Introduction

Type 2 diabetes mellitus (T2DM) is a long-term chronic nutritional metabolic disorder disease that is characterized by high blood sugar, relative lack of insulin, and insulin resistance (1). The mechanism of diabetes is complex due to a variety of factors such as host genetic predisposition, diet, lifestyle changes, and different disease states (2). Oral nutritional (ON) formula is intended for patients with a limited or impaired capacity to intake, digest, absorb, metabolize, or excrete ordinary foods or certain nutrients, or with other medically determined nutrient requirements whose dietary management cannot be achieved by modification of the normal diet. Patients with T2DM have different nutritional requirements compared with normal people. A recent study reported that relatively higher amounts of protein, total fat, monounsaturated fat, and polyunsaturated fat were consumed and relatively lower intakes of carbohydrates, non-milk sugars, and dietary fiber were observed in patients with T2DM compared with normal people (3). The importance of nutritional adjustments in the alleviation of T2DM by influencing weight and regulating metabolism has also been studied (3, 4). In addition, modifying carbohydrate and protein composition, adding monounsaturated fatty acids (MUFA), polyunsaturated fatty acids (PUFA), dietary fiber, and various vitamins and minerals have shown improved blood sugar, lipid, and protein metabolism comparing with a standard diet (5–7).

A diabetes-specific oral formula, which included whey protein (WP), soy protein, multiple carbohydrates, multiple sources of fiber, and various vitamins and minerals could improve postprandial blood glucose (BLG) profiles and 24 h BLG control comparing with a standard-fiber-containing formula based on a randomized, controlled, double-blind, or cross-over study (8). Glycated hemoglobin (HbA1C) was reduced, and hospitalization rates were lowered in patients with T2DM by combining nutritional therapy with other dietary components (9, 10). One of three isoenergetic ready-to-consume formulas showed decreased T2DM symptoms since the short-chain fatty acids in that diet promoted glucagon-like peptide 1 (GLP-1) level (6). Soluble dietary fiber significantly improved postprandial BLG control by delaying glucose absorption in the small intestine (11–13). A very high-protein and low-carbohydrate oral nutrition formula (protein provided 37% of total calories and carbohydrate provided 35% of total calories) also improved BLG with no significant statistical difference in insulin responses on T2DM (5).

L-Arginine, a biological precursor of nitric oxide, has an effect that improves skeletal muscle insulin sensitivity by NO or cGMP pathway to mediate increment of phosphorylation of Akt and AMPK-α (14). It was also found that an average daily intake of more than 800 IU of vitamin-D and more than 1,200 mg of calcium were inversely related to the incidence rate of T2DM when comparing with an intake of <400 IU of vitamin-D and <600 mg of calcium (15). In addition, prebiotics are non-digestible food ingredients that could improve host health by stimulating changes in the composition or activity of specific bacteria in the gut.

Insulin can increase the level of high-density lipoprotein cholesterol (HDL-C) and help people with T2DM to control blood sugar according to 20 randomized controlled trials with 607 adult participants (16). Researchers at Cornell University fed diabetic rats daily with *Lactobacillus rhamnosus* that were engineered to secrete GLP-1. Their results showed that GLP-1 secreting *lactobacilli* could increase plasma insulin levels and glucose tolerance in diabetic rats. Additionally, these rats developed insulin-producing pancreatic cells within the upper intestines up to about 25–33% of the insulin capacity of non-diabetic healthy rats (17).

**Figure 1:**
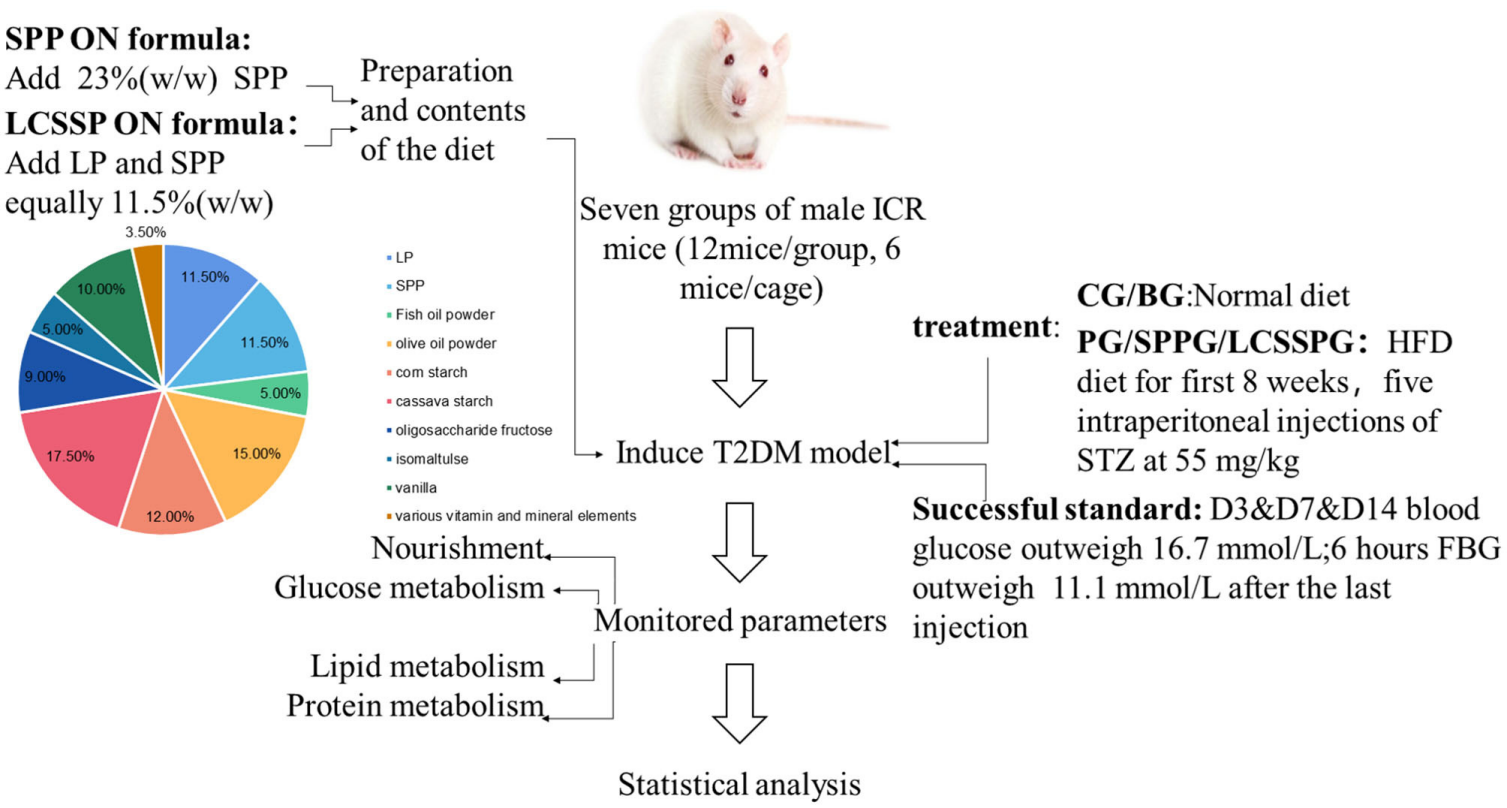
The scheme graph of the whole project.

The effects of various vitamins, minerals, proteins, dietary fiber, MUFA, PUFA, probiotics, prebiotics, and proteins, alone, have been investigated for several years. However, the use of different proteins as the variable in different ON formula *in vivo* was seldom studied. Since protein is an important bioactive molecule, protein may have a key role in the ON formula. Four formula with different proteins as the only difference between the diets were prepared. The three different proteins were soybean (SP), LP, and silkworm pupa protein (SPP) and a combination of LP and SPP. Lipid metabolite indices of plasma, protein metabolite index of plasma, postprandial plasma glucose, and body weight were evaluated. The scheme graph was shown in Figure 1. Results showed that the concentration of total protein (TP) and albumin (ALB) of the plasma was increased in SPG, SPPG, and PG comparing with BG (*p* < 0.05). The TC, TG, and LDL-C levels were decreased, and HDL-C level was increased in SPG, PG, SPPG, PG comparing with BG (*p* < 0.05). BLG levels were decreased 47, 34, 24, and 21% in SPG, LCSSPG, SPPG, and PG, respectively, while BLG was not significantly changed (*p* ≥ 0.05) in LG after 5 weeks of treatment. Overall, the data suggested that consumption of SP, SPP, LCSSPG oral formula may be beneficial for the treatment of T2DM.

## Materials and Methods

### Materials

Streptozotocin (STZ, Sigma Chemical St. Louis, MO, USA), chloral hydrate, sterilized saline water, citric acid, and sodium citrate (Sinopharm Chemical Reagent Co. Ltd., Shanghai, China) were purchased.

### Preparation and Contents of the ON Formula

Three proteins, namely, SP, LP, and SPP were added as 23% (w/w) in SP ON formula, LP ON formula, and SPP ON formula, respectively. The proteins of LP and SPP were equally added (11.5% w/w of each) in the LCSSP ON formula. The rest of the formula was made up of 5% fish oil powder, 15% olive oil powder, 12% corn starch, 17.5% cassava starch, 9% oligosaccharide fructose, 5% isomaltose, 10% vanilla, 3.5% various vitamin, and mineral elements (Table 1). All raw materials for the formula were food-grade and purchased from Henan Jianjiu Industrial Co. (Zhengzhou, Henan, China). Formula tableting was done using a tablet machine (Mini PRESS-IISF, India).

**Table 1:** The enteral formula design of four special protein.

**Nutrient component**	**Content(g/100g)**			
	**SP**	**LP**	**LCSPP**	**SPP**
**Protein**				
Silkworm pupa Protein	0.0	0.0	11.50 ± 1%	23.0 ± 2%
Whey protein	0.0	23.0 ± 2%	11.50 ± 1%	0.0
Soybean protein	23.0 ± 2%	0.0	0.0	0.0
**Fat**				
Fish oil	5.0 ± 1%	5.0 ± 1%	5.0 ± 1%	5.0 ± 1%
Olive oil	15.0 ± 1%	15.0 ± 1%	15.0 ± 1%	15.0 ± 1%
**Carbohydrate**				
Tapioca starch	17.50 ± 1%	17.50 ± 2%	17.50 ± 2%	17.5 ± 2%
Isomaltulose	5.0 ± 2%	5.0 ± 2%	5.0 ± 2%	5.0 ± 2%
Oligosaccharide fructose	9.00 ± 2%	9.00 ± 2%	9.0 ± 2%	9.0 ± 2%
Corn starch	12.0 ± 2%	12.0 ± 2%	12.0 ± 2%	12.0 ± 2%
**Vitamin and Mineral**	Meet the standard of GB-2992-201318		
Dietary fiber	10.00 ± 1%	10.00 ± 1%	10.00 ± 1%	10.00 ± 1%
Essence	2.00×10^−1^	2.00×10^−1^	2.00×10^−1^	2.00×10^−1^

### Nutritional Analysis of the Formula

The methods and standards used to determine the component of the formula were listed in Table 2.

**Table 2:** The methods and standards for the analysis of enteral formula's nutrient composition.

**Nutrient component**	**Measures**	**Standards**
Protein	Kjeldahl (factor: 6.25)	GB50095-2010
Fat	Soxhlet extraction	GB/T 5009.6-2003
Carbohydrate	High performance liquid chromatograph	GB/T 8885-2017GB5009.8-2016
Minerals	Inductively coupled plasma mass spectrometry	GB5009.268-2016
Sodium	mass spectrometry	GB 5009.91-2017
Potassium	mass spectrometry	GB 5009.91-2017
Iodine	Gas chromatography	GB 5009.267-2016
Vitamin		
Choline chloride	spectrophotometry	GB/T 17481-1998
Inositol D-quiral	Gas chromatography	GB5009.270-2016
VB3	plasma mass spectrometry	GB 5009.89-2016
Thiamine	High performance liquid chromatography	GB 5009.84-2016
Riboflavina	High performance liquid chromatography	GB 5009.85-2016
Pyridoxine Hydrochloride	High performance liquid chromatography	GB 5009.154-2016
Cyanocobalamin	High performance liquid chromatography	GB/T 17819-2017
Ascorbic acid	High performance liquid chromatography	GB5009. 86-2016
Palmitic acid VA	colorimetric	GB 5009.82-2016
VD3, cholecalciferol	High performance liquid chromatography	GB 5009.82-2016
Vitamin E	colorimetric	GB 5009.82-2016
Menaquinone	High performance liquid chromatography	GB 5009.158-2016
VB3	High performance liquid chromatography	GB/T 17813-2018
Calcium pantothenate	High performance liquid chromatography	GB 5009.210-2016
VH	spectrophotometry	GB 5009.259-2016
VB9	High performance liquid chromatography	GB/T 17813-2018
L-Arginine	infrared spectroscopy	GB 28306-2012
Taurine	High performance liquid chromatography	GB 5009.169-2016

### Experiment Design

#### Animals

A total of 84 specific-pathogen-free male (SPF) CD-1® (ICR) IGS mice, weighing 18–23 g (aged 6 weeks) were purchased from Beijing Vital River Laboratory Animal Technology Co. Ltd. (Beijing, China). The animal room was maintained at an ambient temperature of 25 ± 4°C, relative humidity of 50 ± 15%, and a light/dark cycle of 12 h (fluorescent light). At the beginning of the experiment, free access to normal rodent chow (20 kJ/kg, 5% fat, 54% carbohydrate, 18% protein, purchased from Shanghai Jiesijie Experimental Animal Co. Ltd., Shanghai, China) and water for the first week was done to give time for acclimatization. The animal study followed ARRIVE (Animal Research: Reporting *in vivo* Experiments) guidelines and was approved by The Institutional Animal Care and Use Committee (IACUC), Shanghai Ocean University Center for Animal Experiment (Shanghai, China).

#### Grouping and Diabetes Modeling

The 84 mice were randomly divided into 7 groups (*n* = 12/group, 6 mice/cage) which included a control group (CG, normal mice, normal food), a non-treated group (BG, diabetic mice, normal food), a positive control group (PG, diabetic mice, HFD), soybean protein group (SPG, diabetic mice, HFD), silkworm pupa protein group (SPPG, diabetic mice, HFD), Whey protein group (LPG, diabetic mice, HFD), and Whey protein combined with silkworm pupa protein group (LCSSPG, diabetic mice, HFD) (Table 3). The HFD (D12451, 47.3 kcal/100g, 45% fat, 35% carbohydrate, 20% protein) was purchased from Suzhou Shuangshi Experimental Animal Feed Technology Co. Ltd., (Suzhou, Jiangsu, China). All the groups were maintained on their diets for 8 weeks.

**Table 3:** Information's about the seven groups.

**Groups**	**Groups**	**Nutrition support**	**STZ injection**
BG	Blank group	Normal diet	Yes
PG	Positive control group	Abbott glucerna Slow Release powder	Yes
LCSSPG	Lactoalbumin Combined Silkworm Pupa Protein group	Lactoalbumin Combined Silkworm Pupa protein ON tablets	Yes
SPPG	Silkworm pupa protein group	Silkworm pupa protein ON tables	Yes
LPG	Lactoalbumin protein group	Lactoalbumin protein ON tablets	Yes
SPG	Soybean protein group	Soybean protein ON tablets	Yes
CG	Control group	Normal diets	No

The study was conducted according to the method of Sun et al. (18). After 8 weeks, the mice fed with HFD were intraperitoneally given an injection of 55 mg/kg body weight STZ. Thereafter, they were given additional injections at the same time every other day for four additional shots. The STZ powder was dissolved immediately into 0.1 mol/L citrate acid buffers (pH 4.5) in an ice bath for 20 min (19). The CG mice were given an injection of an equivalent volume of the citric acid buffer. Mice were considered diabetic when at 3-, 7-, and 14-days BLG exceeded 16.7 mmol/L (20), and 6 h fasting BLG exceeded 11.1 mmol/L after the last injection (21). Twenty days after the first STZ shot, the feeding of the ON formula began. The BLG was determined using a BLG meter (Sannuo GA-3 type, Changsha, Hunan, China). All mice had free access to diet and water.

#### Measurements

##### Body Weight and Other Animal Measurements

Body weight was measured every week from the beginning of the experiment. The mice were sacrificed with chloral hydrate at 4.5 mg per 100 g body weight, and blood was drawn from the heart. One mouse per group was fasted for 6 h and sacrificed at weeks 2, 10, 11, and 14. All remaining mice were sacrificed by week 15. Approximately 1 ml of blood was obtained from each mouse in a 2 ml sterile citrate anticoagulant centrifuge tube and the plasma was separated by centrifugation at 4,000 g/min, for 15 min at 4°C. The serum was separated and stored at −80°C for further analysis, a maximum of 1 year (22).

##### Lipid Metabolite Indices of Plasma

Total cholesterol (TC), triglycerides (TG), low-density lipoprotein cholesterol (LDL-C), and HDL-C levels were measured using an automatic biochemical analyzer (AU5800 Clinical Chemistry System, Beckman Coulter, S.Kraemer Boulevard Brea, CA92821, USA).

##### Protein Metabolite Index of Plasma

The levels of TP and ALB were detected in the plasma of experimental animals using an automatic biochemical analyzer (AU5800 Clinical Chemistry System, Beckman Coulter, S. Kraemer Boulevard Brea, CA92821, USA).

##### Glucose Metabolite Index of Plasma

The fasting blood glucose (FBG) level was measured every week. Postprandial plasma glucose was measured at weeks 9, 11, 13, and 15 after mouse feeding. Blood samples were collected from the tail vein and BLG levels were determined using the glucometer.

### Statistical Analysis

All data were analyzed as the mean ± SD and levels were compared among groups using one-way ANOVA. Statistical analysis was done using the Statistical Program for the Social Sciences Version 23.0 (SPSS, Chicago, IL, USA). Line chart and histogram were prepared using origin pro-2019 (Wellesley Hills, MA, USA). Differences were taken as significantly different when *p* < 0.05 and were considered very significant when *p* < 0.01.

## Results and Discussion

### The Composition of Four ON Formula

The formulations of four kinds of the ON formula were analyzed according to the methods in Table 2 and listed in Table 4. The nutrient compositions of the four ON formula were analyzed in SPG, LPG, LCSSPG, and SPPG. The protein contents were 19.9, 20.1, 21.3, and 21.8 g/100 g, respectively. Carbohydrate contents were 43, 42.9, 43.6, and 42.1 g/100 g, respectively. Fat contents were 19.1, 19.3, 19.5, and 19.6 g/100g, respectively. The total energy of four ON formula was lower than 1870.5KJ. The fat-supplied energy was less than 35%, carbohydrate-supplied energy was less than 45%, and the proportion of protein-supplied energy was less than 23% in all self-made oral formula.

**Table 4:** Formulations of enteral nutritional formula.

**Nutrient component**	**The Content(g/100g)**			
	**SPG**	**LPG**	**LCSSPG**	**SPPG**
Protein	19.9 ± 0.4	20.1 ± 0.1	21.3 ± 0.3	21.8 ± 0.6
Fat	19.1 ± 0.22	19.3 ± 0.1	19.6 ± 0.5	19.6 ± 0.2
Carbohydrate	43.0 ± 0.1	42.9 ± 0.1	43.5 ± 0.4	42.1 ± 0.3
Mineral				
Sodium	0.41 ± 0.03	0.41 ± 0.02	0.38 ± 0.02	0.45 ± 0.1
Potassium	0.99 ± 0.2	0.94 ± 0.1	0.94 ± 0.1	0.95 ± 0.1
Chloride	0.78 ± 0.1	0.71 ± 0.2	0.78 ± 0.2	0.77 ± 0.2
Calcium	0.4 ± 0.04	0.38 ± 0.07	0.41 ± 0.4	0.42 ± 0.1
Phosphate	0.54 ± 0.05	0.51 ± 0.11	0.5 ± 0.1	0.52 ± 0.2
Magnesium	(8. ± 0.4)×10^−4^	(8.1 ± 0.1×10^−4^	(8.3 ± 0.1)×10^−4^	(8.2 ± 0.2)×10^−4^
Ferric	(1.3 ± 0.1)×10^−2^	(1.5 ± 0.3)×10^−2^	(1.7 ± 0.2)×10^−2^	2.0 ± 0.2)×10^−2^
Zinc	6.8 ± 0.4)×10^−3^	(7.0 ± 0.2)×10^−3^	(7.1 ± 0.2)×10^−3^	(6.9 ± 0.2)×10^−3^
Copper	(8.5 ± 0.3)×10^−4^	(8.7 ± 0.2)×10^−4^	(8.3 ± 0.24)×10^−4^	(8.8 ± 0.1)×10^−4^
Manganese	(6.8 ± 0.2)×10^−4^	(6.6 ± 0.1)×10^−4^	(6.5 ± 0.1)×10^−4^	(6.5 ± 0.1)×10^−4^
Molybdenum	(7.1 ± 0.3)×10^−4^	(7 ± 0.2)×10^−4^	(6.9 ± 0.3)×10^−4^	(7.2 ± 0.2)×10^−4^
Selenium	(1.9 ± 0.2)×10^−4^	(1.8 ± 0.1)×10^−4^	(2 ± 0.2)×10^−4^	(2 ± 0.1)×10^−4^
Vitamin				
Choline chloride	0.16 ± 0.03	0.14 ± 0.01	0.16 ± 0.2	0.14 ± 0.1
Inositol D-quiral	0.3 ± 0.04	0.32 ± 0.02	0.33 ± 0.1	0.2 ± 0.1
Tiamina	(8.0 ± 0.25)×10^−4^	(6.0 ± 0.1)×10^−4^	(7.0 ± 0.4)×10^−4^	(6.0 ± 0.2)×10^−4^
Riboflavina	(3.0 ± 0.4)×10^−4^	(4.0 ± 0.2)×10^−4^	(5.0 ± 0.3)×10^−4^	(5.0 ± 0.4)×10^−4^
Pyridoxine Hydrochloride	(1.1 ± 0.2)×10^−3^	(1 ± 0.2)×10^−3^	(1.3 ± 0.2)×10^−3^	(1.2 ± 0.2)×10^−3^
Ascorbic acid	(3.0 ± 0.5)×10^−2^	(4.0 ± 0.1)×10^−2^	(3.5 ± 0.3)×10^−2^	(3.8 ± 0.1)×10^−2^
Palmitic acid VA	(5.0 ± 0.3)×10^−4^	(6 ± 0.2)×10^−4^	(9.0 ± 0.1)×10^−4^	(8.0 ± 0.2)×10^−4^
VD3 cholecalciferol	(1.2 ± 0.3)×10^−5^	(1.6 ± 0.2)×10^−5^	(1.1 ± 0.1)×10^−5^	(1.5 ± 0.2)×10^−5^
Vitamin E	(2 ± 0.3)×10^−3^	(2.6 ± 0.1)×10^−3^	(2.3 ± 0.1)×10^−3^	(2.3 ± 0.1)×10^−3^
Phytonadione	(4.0 ± 0.4)×10^−5^	(6.0 ± 0.1)×10^−5^	(9.0 ± 0.2)×10^−5^	7.1 ± 0.2)×10^−5^
VB3	(3.6 ± 0.2)×10^−3^	(3.6 ± 0.1)×10^−3^	(2.8 ± 0.1)×10^−3^	(3.0 ± 0.1)×10^−3^
Calcium pantothenate	(2.4 ± 0.2)×10^−3^	(2.1 ± 0.1)×10^−3^	(2.2 ± 0.1)×10^−3^	(2.0 ± 0.1)×10^−3^
VH	(3 ± 0.2)×10^−4^	(3.3 ± 0.1)×10^−4^	(3.1 ± 0.1)×10^−4^	(3.2 ± 0.2)×10^−4^
VB9	(1 ± 0.3)×10^−4^	(1.2 ± 0.2)×10^−4^	(1.1 × 10-3)×10^−4^	(1.1 ± 0.1)×10^−4^
L-arginine	0.2 ± 0.03	0.21 ± 0.04	0.22 ± 0.1	0.21 ± 0.1
Taurine	(3. ± 0.5)×10^−2^	(3.4. ± 0.2)×10^−2^	(3.9 ± 0.2)×10^−2^	(3.1 ± 0.1)×10^−2^

The compositions of the four ON formula were similar and protein type was the only way to distinguish them. The differences among those three proteins were, mainly, amino acid composition. Eighteen kinds of amino acids were detected in SP which included 1.8 g of methionine, 6.1 g of lysine 1.2 g of cysteine, 4.0 g of threonine, and 4.6 g/16 g N of alanine on average and so on (23). Stefan et al. demonstrated that the essential amino acids content of SP and LP was 27% and 43%, respectively (24). SPP, as a novel high-quality raw material for complementary medicine food and the mass ratio of essential or non-essential amino acids, was.77, which was higher than the FAO-WHO requirements (>0.6) (25).

Relatively lower essential amino acids content led to better protein metabolism according to the results. Nonetheless, the amino acid mixtures cannot reach identical results comparing with protein consumption (26). Plasma amino acid, closely related to muscle protein synthesis, was increased after protein intake (24, 25).

Diet can also lower the rate of developing diabetes in those with impaired glucose tolerance (7, 27, 28). Proper amounts of vitamins and minerals which include zinc, chromium, magnesium, vitamin B group, and inositol were added to alleviate glucose and lipid metabolism disorder in T2DM mice (28). An amount of 10 g/100 g dietary fiber was supplemented in the ON formula to prolong gastric emptying time and delay glucose absorption. A series of systematic reviews and meta-analyses suggested that the T2DM rate was decreased 15-30% when there is a 25–29 g dietary fiber intake according to a 2019 review report (29). Tapioca starch was added to the ON formula to stable postprandial BLG (30).

Fish oil- and olive oil-supplemented diets provided better glycaemic and lipid metabolic control in both human and animal experiments comparing with a normal diet. Eight grams of olive oil per day has a 5% T2DM risk-reducing effect according to a 22-year follow-up study (31). David et al. showed that an HFD, supplemented with fish oil for 30 weeks lowered the effects of liver cholesterol, cholesterol ester, and triacylglycerol in C57BL/6J mice compared with mice fed an HFD supplemented with lard (32). In this present study, the self-made ON formula combined all the features above.

### The Regulation of Protein Metabolism by Four Oral Formula *in vivo*

The TP, ALB, and GLO levels in serum were analyzed to explore whether ON formula supplemented with specifically designed proteins had an effect on protein metabolism (Table 5). The TP, ALB, and ALB/GLO levels were decreased in BG when compared with CG. The concentration of TP in LCSSPG, SPPG, and SPG was decreased to 31, 39, and 39.5 mmol/L, respectively, which nearly reached normal levels after 4 weeks. The ALB level was significantly increased from 15.0 to 22 and 22.5 in SPPG and SPG, respectively. The TP, ALB, and GLO levels were increased in PG (*p* < 0.05) while no statistically significant difference in the ratio of ALB/GLO level was observed.

**Table 5:** Effect of different enteral formula on TP, ALB and GLO in high fat diet induced mice.

**Group**	**TP (mmol/L)**	**ALB (mmol/L)**	**GLO (mmol/L)**	**ALB/ GLO (%)**
CG	46.6 ± 2.0^**^	26.5 ± 2.0^**^	20.4 ± 1.1^**^	1.31 ± 0.08^**^
BG	29.5 ± 3.0	15.0 ± 2.0	14.3 ± 0.7	1.07 ± 0.03
PG	32 ± 1.5	16 ± 1.0	13.5 ± 1.7	1.05 ± 0.03
LCSSPG	31.0 ± 2.0	18 ± 0.7	13 ± 0.7	1.35 ± 0.1^*^
SPPG	39.5.0 ± 1^*^	22.5 ± 0.3^*^	17 ± 0.7^*^	1.33 ± 0.03^*^
LPG	26.5 ± 1.0	15.5 ± 0.3	14 ± 0.7	1.11 ± 0.03
SPG	39.5 ± 0.3^*^	22.5 ± 1^*^	16.5 ± 0.3^*^	1.40 ± 0.08^*^

*Data represent as mean ± SEM*.

* *Represents p < 0.05 vs. blank group*.

** *p < 0.01 vs. blank group*.

A decrease in the levels of TP, ALB, and GLO was verified both in T2DM humans and mice which may relate to the factor of insulin resistance in pancreatic β-cells (33). As expected, specially added proteins such as SPP and SP can release functional amino acids which can repair amino acid stimulated protein synthesis to further alleviate whole-body protein metabolism. Taken together, the results demonstrated that ON formula containing suitable protein such as SP or SPP at a proportion of 23 g/100 g could bring back protein levels to normal in plasma comparing with normal diet and even better than available ON formula products.

### The Regulation of Lipid Metabolism by Four Oral Formula

**Figure 2:**
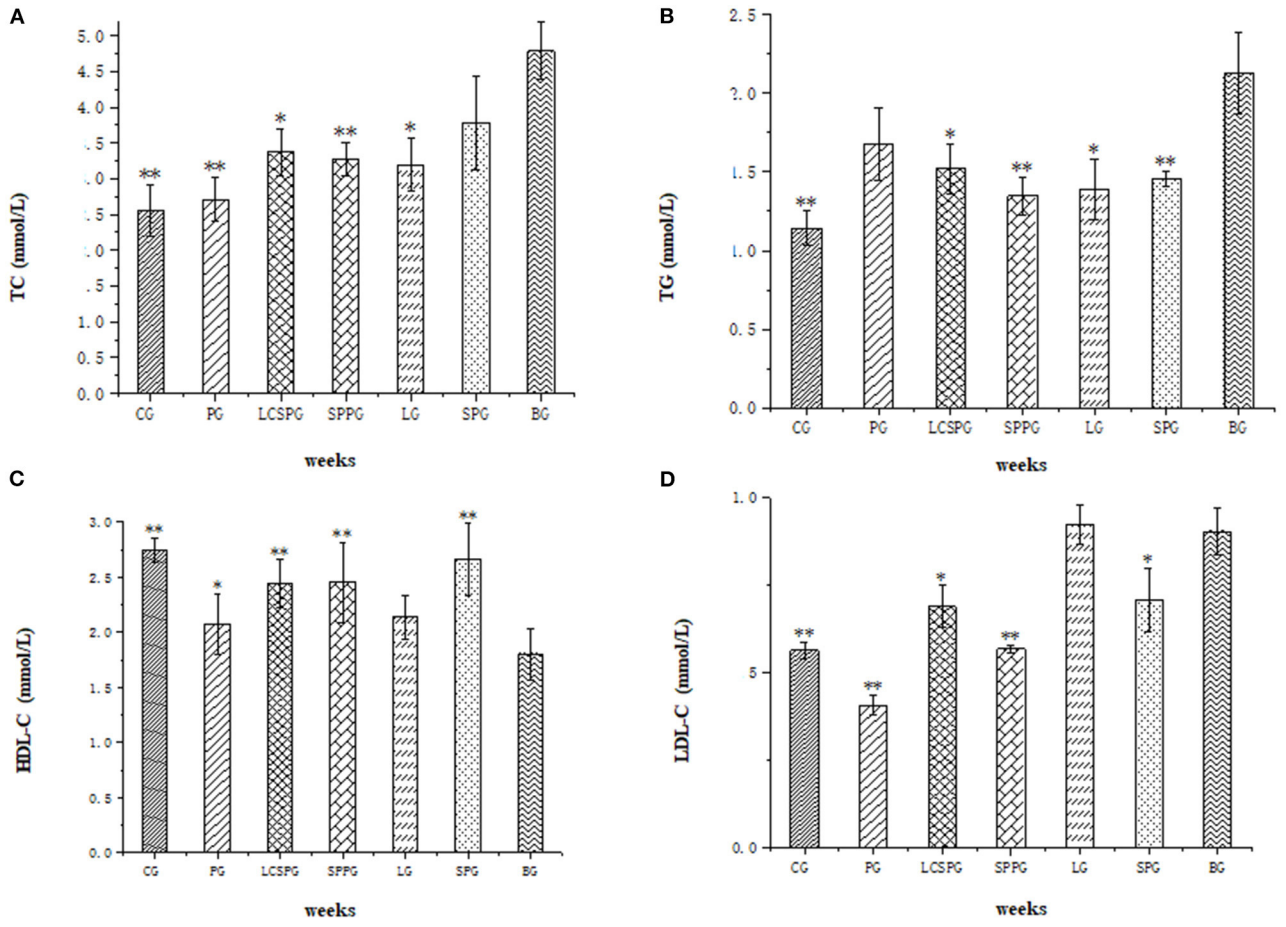
**(A–D)** Total cholesterol concentration (TC). **(A)** triglycerides (TG), **(B)** high-density lipoprotein cholesterol (HDL-C), and **(C)** low-density lipoprotein cholesterol (LDL-C) **(D)** levels of seven groups. Data represented as mean ± SD *p < 0.05 and **p < 0.01: compared with a blank group (BG). BG was T2DM mice fed the normal diet; CG was normal mice fed a normal diet; PG was T2DM mice fed Abbott Glucerna Slow Release; LG was T2DM mice fed with LP ON formula; SPG was T2DM mice fed SP ON formula; SPPG was T2DM mice fed SPP ON formula; LCSSPG was T2DM mice fed LCSSP ON formula.

The effects of ON formula consumption on lipid metabolism were shown in Figure 2. Statistical differences were conducted among seven groups which were shown in Figure 1. The TC, TG, LDL-C were increased significantly after 8 weeks high-fat diet (HFD) combined with STZ intraperitoneal injection. The HDL-C level was significant decreased in the BG comparing with CG (*p* < 0.01). The serum TC level was significantly reduced after consumption of SP, SPP, LCSSPP, PG, and LP comparing with BG (Figure 2A). The TG level was significantly decreased after administration of SP, SPP, LCSPP, and LP comparing with BG (Figure 2B). In the study, it was found that SP, SPP, LCSSP, and LP ON formula had a great role in decreasing the serum TG and TC level in HFD induced mice. The HDL-C levels were increased significantly on LCSSPPG, SPPG, SPG, and PG comparing with the BG group indicating that LCSSPP, SPP, and SP oral formula may lower the risk of HDL-C loss in HFD-induced mice (Figure 2C). The level of serum LDL-C was greatly lowered after consumption of PG, SPPG, LCSSPG, and SPG while there was no significant difference in the LPG group which needed further analysis (Figure 2D). These data indicated that PG, SPPG, LCSSPG, and SPG had a good supporting role in decreasing the serum LDL-C level in HFD induced mice.

This study proved that the ON formula could significantly reduce serum TC, TG, HDL-C, and LDL-C levels in T2DM mice comparing with a normal diet. The results are consistent with previous research reported by Sung et al. (33). The proteins used in the ON formula are highly responsible for those changes in lipid metabolism. Sun He et al. found the anti-obesity activity of a silkworm pupa peptide in an HFD feeding in rats by inhibiting the differentiation of preadipocytes and adipogenesis (33). Rizaldy et al. verified the decreased weight and adiposity of LP in male obese rats due to decreased hepatic lipidosis compared to control, partly through downregulation of lipogenic and upregulation of β-oxidation transcripts in the liver (34). Based on literature reviews, a diet with soy protein containing isoflavones reduced low-density lipoprotein (LDL) (35), but without clear effects on TG or high-density lipoprotein (HDL). The results demonstrated similar results which alleviate lipid metabolism by high-quality protein addition.

### The Regulation of BLG by Four ON Formula

**Figure 3:**
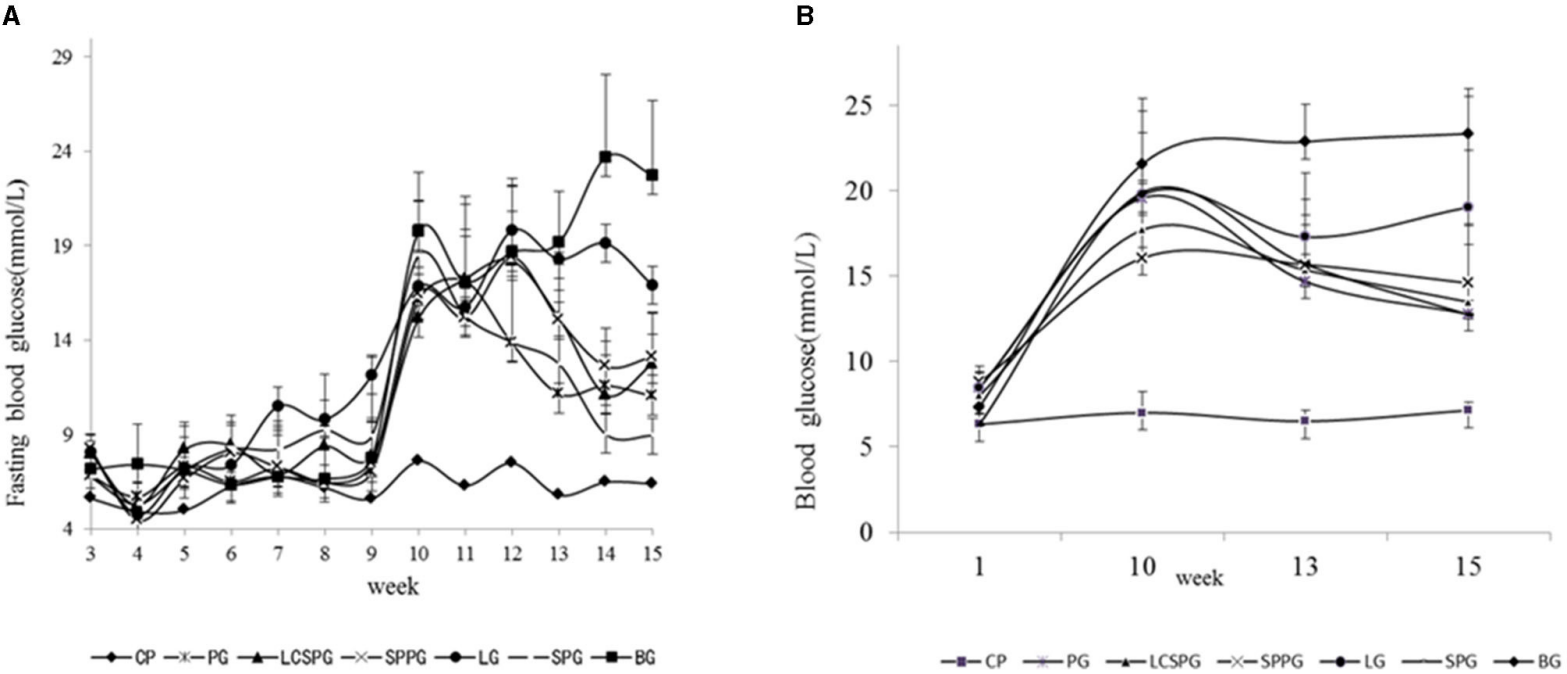
Changes in blood glucose (BLG) **(B)** and fasting BLG **(A)** of the mice fed with a high-fat diet (HFD) combined with an intraperitoneal injection of streptozotocin (STZ) during the experiment.

The BLG and fasting BLG changes during the experiment are shown in Figure 3, respectively. The FBG and BG were enhanced from 7 to 10 wks and peaked in the tenth week. The FBG has a great reduction with the value of 46.7, 24.1, 21.8, and 33.9% in SPG, LCSSPG, SPPG, and PG group, respectively, after 5 wks of treatment. Continuous elevation of FBG and BLG in BG was observed, which is strong evidence for T2DM modeling making. The FBG level was decreased to 16.8 to 8.9, 12.8, 13.2, and 11 in SPG, LCSSPG, SPPG, and PG groups at the end of the experiment. Furthermore, there were no wide fluctuations in BLG levels at the end of treatment, which needed to be further analyzed.

The study, *in vivo*, indicated that the administration of the ON formula to the HFD-fed mice combined with an intraperitoneal injection of STZ produced a remarkable reduction of BLG and FBG after 5 weeks of treatment with SPPG, SPG, and LCSSPG. This was true except for the LPG and PG since they showed a low reduction on these parameters. The main proteins used in the formulations played an important role in the hypoglycaemic effect. Further, another study has shown that soybean supplementation would be helpful to control BLG and serum lipid in patients with diabetes (35). The soybean fiber contains compounds with high viscosity like pectin, galactomannans, and arabinogalactans that delay gastric emptying and glucose absorption. Novels peptides from silkworm pupae were found by Yu et al. (36) with high α-glucosidase-inhibiting activity so that this protein can retard glucose absorption and suppressing postprandial hyperglycaemia.

### The Regulation of Body Weight by Four Oral Formula

**Figure 4:**
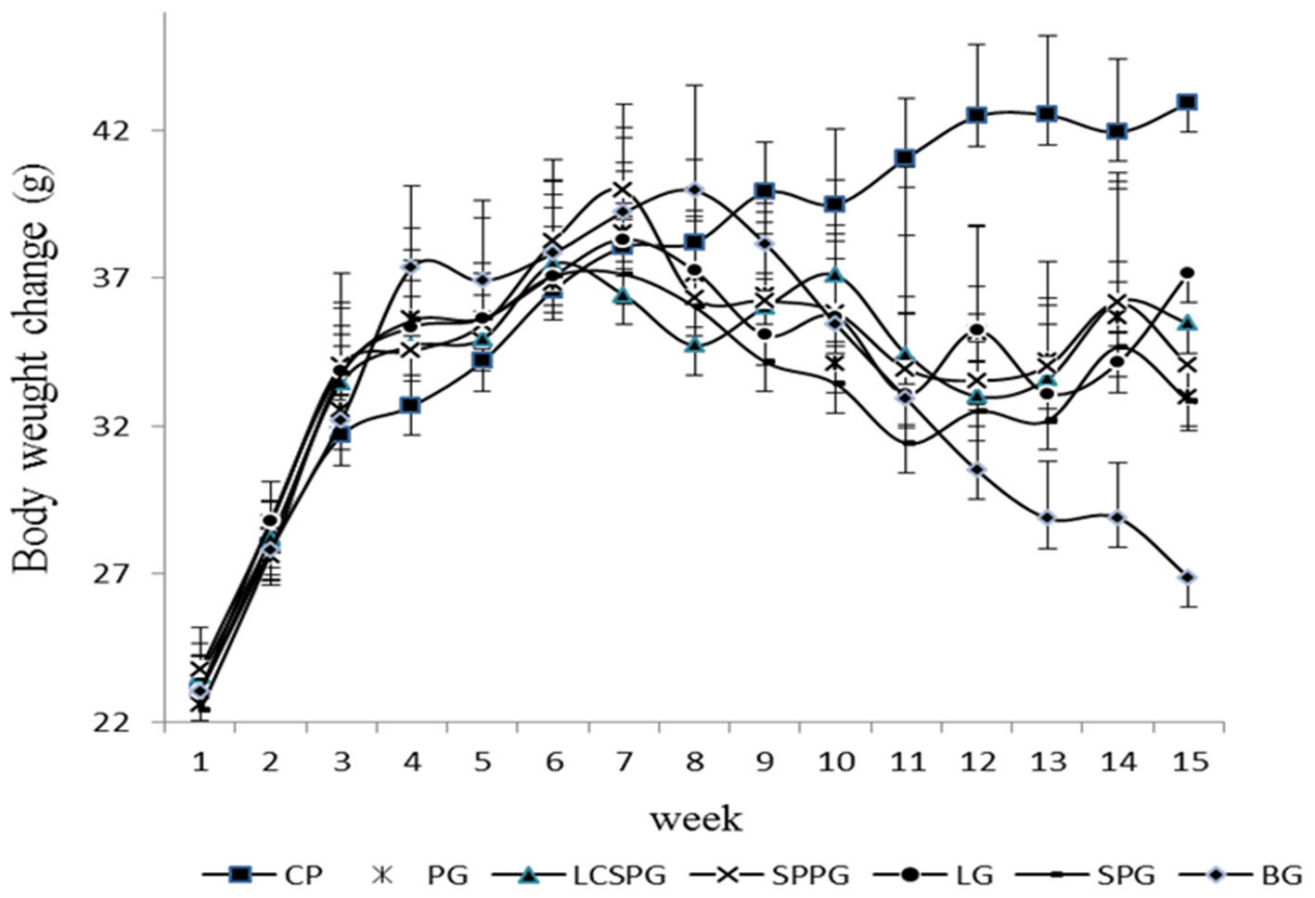
Body weight change of mice when HFD, normal diet, and ON formula was given to corresponding groups for 15 weeks.

The body weight changes after the 15-week feeding in the experiment are shown in Figure 4. Body weight gain rate was similar at the beginning of feeding in the HFD induced group (LPG, SPG, LCSSPG, SPPG, BG, and PG) and their body weight doubled after 8 weeks of HFD feeding. Increased thirst, frequent urination, and unexplained weight loss were the most common symptoms of patients with T2DM (37). The body weight decreased sharply (3.26 to 5.33 g per mouse) after T2DM models were successfully established in the 10th week in SPG, SPPG, LPG, CG, BG, and LCSSPG with no obvious differences among groups, which was consistent with the above mentioned. Body weight increased after 2 weeks of treatment and increased onward but slightly decreased (about 0.8–1.2 g per mice) after 5 weeks of treatment in all groups except CG and LG. Body weight loss was stopped after 2 weeks of treatment and increased onward until the end of treatment in LPG. The body weight loss in BG was continued at the end of the experiment which claimed the successful development of T2DM mice.

Body weight, on behalf of whole-body nutrient metabolism index with losses over 10% could lead to metabolism dysfunction (38). Thus, the degree of weight stability and restoration was taken as a key factor in reversing T2DM symptoms. The body weight declined to the minimum value after 2 weeks of treatment and then plateaued and increased. The body weight gain occurred after a 2-week interval, which was similar to the previous study by Qingbo et al. (25). Over 10% of body weight loss was observed among all groups in the 2-week treatment of minimum weight. Therefore, a suitable amount of ON intervention in time is necessary to inhibit the deterioration of body weight loss. An obvious difference in body weight loss among groups was observed according to statistical analysis. The body weight gain was more rapid in the group fed with ON tablets compared with BG and PG. In the current study, at least a 3-week consumption of ON formula could help to reverse T2DM induced body weight loss according.

## Conclusion

Dietary protein influences lipid, protein, and glucose metabolism (8). SP could lower cholesterol levels in animals and humans with unclear mechanisms (5, 9). Moriyama et al. suggested that the soybean β-conglycinin diet could reduce serum TG and glucose in genetically obese mice (37). The high content-release of essential amino acids, such as leucine, isoleucine, valine, lysine, and threonine, by LP after digestion, lead to increased secretion of insulin and decrease postprandial hyperglycaemia was verified (34). Emilia et al. pointed that branched short-chain fatty acids (isobutyric, isoleucine, and isovaleric) have an impact on glucose metabolism by improving insulin sensitivity with disturbed metabolism (39). LP can also promote the secretion of GLP-1 and gastric inhibitory peptide (GIP).

The GLP-1a incretin hormones, which can promote glucose-dependent insulin secretion, are the main drug targets in T2DM at present. Meanwhile, GIP is released from K cells after food intake. A greater weight gain, insulin resistance, and hepatic steatosis emerged in the HFD fed mice with GIP receptor deficiency (40). The mass ratio of essential amino acid to non-essential amino acid is superior to the reference mode proposed by FAO-WHO (41).

In this study, the effect of three different oral formula on T2DM *in vivo* was evaluated by some lipid, protein, and glucose metabolite index of plasma. Results showed that levels of TP, ALB, GLO, HDL-C, and ALB/GLO ratio were increased while levels of FBG, BLG, LDL-C, TC, and TG were decreased, all of which were associated with alleviating symptoms after administration of ON formula to SPG, SPPG, LCSSPG, and PG. Body weight, on behalf of whole-body nutrient metabolism, showed a significant different increasing trend between the treatment group and BG. Also, SP and SPP ON formula showed a greater alleviating effect comparing with another treatment group after 5 weeks of treatment. These specifically designed proteins are likely the key role for the restoration of T2DM in ON formula by enhancing a beneficial function and coinciding with the improvement of whole-body nutrient metabolism. Therefore, ON formula may present a novel nutrition therapeutic strategy for the treatment of T2DM.

## Data Availability Statement

The raw data supporting the conclusions of this article will be made available by the authors, without undue reservation.

## Ethics Statement

The animal study was reviewed and approved by Shanghai Ocean University Center for Animal Experiment (Shanghai, China).

## Author Contributions

PW and MM revising it critically and supporting this study. AC and YL carrying out the animal lab work and participating in data analysis. BB and WW design of the study and for drafting the manuscript partially. CB collecting field data. All the authors gave final approval for publication.

## Funding

This study was supported by the Innovation Action in Shanghai (No. 1749074222500), the National Natural Science Foundation of China (No. 81502955, 81750110548), and Xinjiang Production and Construction Corps Key Laboratory of Protection and Utilization of Biological Resources in Tarim Basin (No. BRZD1904).

## Conflict of Interest

The authors declare that the research was conducted in the absence of any commercial or financial relationships that could be construed as a potential conflict of interest.

## Publisher's Note

All claims expressed in this article are solely those of the authors and do not necessarily represent those of their affiliated organizations, or those of the publisher, the editors and the reviewers. Any product that may be evaluated in this article, or claim that may be made by its manufacturer, is not guaranteed or endorsed by the publisher.
